# Intrinsic connectomes are a predictive biomarker of remission in major depressive disorder

**DOI:** 10.1038/s41380-019-0574-2

**Published:** 2019-11-06

**Authors:** Mayuresh S. Korgaonkar, Andrea N. Goldstein-Piekarski, Alexander Fornito, Leanne M. Williams

**Affiliations:** 1 The Brain Dynamics Centre, Westmead Institute for Medical Research, The University of Sydney, Sydney, Australia; 2 Discipline of Psychiatry, Western Clinical School, The University of Sydney, Sydney, Australia; 3 Department of Psychiatry and Behavioral Sciences, Stanford University, Stanford, California USA; 4 Sierra-Pacific Mental Illness Research, Education, and Clinical Center (MIRECC) VA Palo Alto Health Care System, Palo Alto, CA USA; 5 Brain and Mental Health Research Hub, Turner Institute for Brain and Mental Health & Monash Biomedical Imaging, Monash University, Clayton, Victoria Australia

**Keywords:** Prognostic markers, Depression

## Abstract

Although major depressive disorder (MDD) is associated with altered functional coupling between disparate neural networks, the degree to which such measures are ameliorated by antidepressant treatment is unclear. It is also unclear whether functional connectivity can be used as a predictive biomarker of treatment response. Here, we used whole-brain functional connectivity analysis to identify neural signatures of remission following antidepressant treatment, and to identify connectomic predictors of treatment response. 163 MDD and 62 healthy individuals underwent functional MRI during pre-treatment baseline and 8-week follow-up sessions. Patients were randomized to escitalopram, sertraline or venlafaxine-XR antidepressants and assessed at follow-up for remission. Baseline measures of intrinsic functional connectivity between each pair of 333 regions were analyzed to identify pre-treatment connectomic features that distinguish remitters from non-remitters. We then interrogated these connectomic differences to determine if they changed post-treatment, distinguished patients from controls, and were modulated by medication type. Irrespective of medication type, remitters were distinguished from non-remitters by greater connectivity within the default mode network (DMN); specifically, between the DMN, fronto-parietal and somatomotor networks, the DMN and visual, limbic, auditory and ventral attention networks, and between the fronto-parietal and somatomotor networks with cingulo-opercular and dorsal attention networks. This baseline hypo-connectivity for non-remitters also distinguished them from controls and increased following treatment. In contrast, connectivity for remitters was higher than controls at baseline and also following remission, suggesting a trait-like connectomic characteristic. Increased functional connectivity within and between large-scale intrinsic brain networks may characterize acute recovery with antidepressants in depression.

## Introduction

Antidepressant medications remain the first-line treatment for major depressive disorders (MDD) [1, 2], yet more than 50% of patients fail to achieve remission upon initial treatment [3, 4]. Failure to respond to first-line treatments not only lengthens recovery time, but also reduces response rates to subsequent medications, contributing to the enormous burden of disease to the patient, family, health systems and society [5]. Understanding neurobiological mechanisms of antidepressant action is crucial to elucidating the characteristics of favorable treatment outcome. Intensive research using advanced neuroimaging techniques over the past few decades has shed light on the neurobiological substrates of depression [6]. However, our understanding of the neural mechanisms through which some people recover on antidepressants and some people do not is still rudimentary.

The human brain is intrinsically organized into distinct, functionally coherent networks, whose synchrony underlies cognitive and emotional processes [7]. MDD has been characterized by abnormal interactions within and between these intrinsic brain networks, particularly those that regulate awareness of internal states (i.e., the default mode network, DMN), external awareness (the dorsal attention network, DAN), those involved in top-down regulation of attention and emotion (fronto-parietal network, FPN; and cingulopercular network, CON); and those involved in processing of emotion (affective network, AN) and monitoring for salient events (ventral attention network, VAN). Specific patterns of aberrant communication between these networks are known to contribute to core cognitive and affective deficits in MDD [8].

Increasing attention is being paid to understanding the effects that antidepressant medications exert on brain connectivity in MDD, as measured using either task-free or task-based fMRI (reviewed recently in refs. [9, 10]). Treatment with antidepressants have consistently found to alter functional connectivity of the DMN [11] and cortico-limbic structures [12, 13], with little effect on that within the cognitive control network (FPN) [14]. However, there are few existing imaging studies of antidepressant treatment response and these have mainly examined limited sample sizes [12, 15–18]. The majority of these studies have also employed a pre-defined seed based connectivity analysis, which only considers a small subset of the brain [11, 12, 18]. Although such a focused approach is useful to examine specific brain networks or specific neural connections, it limits a holistic and integrated systems level understanding of how these treatments affect the brain and its intrinsic functional networks. Finally, the primary focus for these previous studies has been on understanding the impact of antidepressants on neural changes and how these relate to changes in symptoms. Few studies have examined whether pre-treatment intrinsic neural characteristics may pre-dispose an individual to respond to antidepressant medications [19, 20]. Based on previous studies using both antidepressant medications and non-pharmaceutical treatments, there is some evidence to suggest that pre-treatment intrinsic brain connectivity, particularly related to the DMN, is associated with response to these treatments [19–21].

Here, we adopted a comprehensive, connectome-wide approach [22, 23] to investigate large-scale intrinsic functional brain networks that characterize remission to antidepressant medications prior to treatment in a cohort of 163 MDD patients. We analyzed intrinsic functional connectivity from fMRI scans collected prior to and following an 8-week course of randomly assigned one of three commonly prescribed first-line antidepressants in a practical clinical trial design. We also examined whether connectivity within the remission-related brain networks at baseline differs between MDD participants and healthy participants, whether it changes with treatment generally, and as a function of the specific medication used. Based on previous evidence, we hypothesized that pre-treatment functional connectivity related to the DMN is likely to characterize remitters from non-remitters, to differ between depressed and healthy individuals, and that DMN connectivity will also change following treatment. Our connectome-wide approach offers a powerful, comprehensive, and regionally unbiased way of examining the specificity of DMN connectivity.

## Materials and methods

### Participants and study protocol

All participants that completed both the baseline and 8-week post-treatment fMRI scans from the imaging cohort of the iSPOT-D study were included in this analysis [24, 25]. Data were available for 163 MDD participants (out of the 204 that were recruited at baseline) and 62 age- and gender-matched healthy participants (CONSORT chart provided in Supplementary Fig. S1). The iSPOT-D study protocol, clinical assessments and inclusion/exclusion criteria have been previously described [25]. In short, the Mini-International Neuropsychiatric Interview [26], according to DSM-IV criteria, and a 17-item Hamilton Rating Scale for Depression (HRSD_17_) [27] score ≥16 confirmed the primary diagnosis of MDD. At baseline, all MDD participants were either antidepressant naïve or had undergone a washout period of at least five half-lives of a previously prescribed antidepressant medication. Healthy control participants were extensively screened for the absence of Axis I disorders and for an HRSD_17_ score less than or equal to 7. MDD participants were randomized to receive flexibly dosed, open-label escitalopram, sertraline or venlafaxine-extended release (venlafaxine-XR) for 8 weeks at the end of which they completed the follow-up MRI session. Our study recruited from primary care, community, and academic psychiatry settings with the goal of representing a broad sample of antidepressant treatment seekers. Medications were prescribed and doses adjusted by treating clinicians according to routine clinical practice but following the recommended dose ranges. An HRSD_17_ of ≤7 was used to define remission at week 8 (MDD-R: remitters and MDD-NR: non-remitters). In addition to the HRSD_17_ score, participant age, gender, age of onset of depression, depression duration, number of previous depression episodes, previous treatment, melancholia, score of the 42 item depression-anxiety-stress scale (DASS) [28] were recorded. Sample size was chosen as part of the original protocol development in order to achieve statistical power of 80% at an effect size of 1 standard deviation [24]. Participants provided written informed consent in accordance with the ethical guidelines of the institutional review board (Western Sydney Local Health District Human Research Ethics Committee).

### fMRI acquisition, pre-processing, and generation of functional connectomes

Details of MRI acquisition, activation tasks, pre-processing, and intrinsic connectivity estimation were published previously [20, 24, 29] and can be found in Supplementary Section S1. In brief, MRI data for both visits were acquired on a 3T GE Signa HDx scanner using an 8-channel head coil. MRI acquisition included five fMRI tasks (echo planar imaging; TR/TE = 2500/27.5 ms, Flip Angle = 90°, 64 × 64 matrix, 40 axial 3.5 mm slices, 120 volumes) and a 3D T1-weighted structural MRI scan (TR/TE = 8.3/3.2 ms, Flip Angle = 11°, TI = 500 ms, 256 × 256 matrix, 180 sagittal 1 mm slices). Intrinsic functional connectivity was estimated using data from all five tasks. fMRI images were motion-corrected and corrected for geometric distortions using realignment and unwarping, slice time corrected, spatially normalized to the stereotactic MNI space and smoothed. As motion is a critical issue in resting state data, data volumes associated with high movement (framewise displacement from one time point to the next) or changes in BOLD signal intensity were censored (temporally masked) to reduce the influence of motion and related artifacts [30, 31]. For each fMRI task, the BOLD responses for each experimental condition were modeled in the general linear model framework. Additional covariates for each task included the mean signal time course extracted from eroded ventricle and white matter masks, as well as the temporal masks derived from the volume censoring described above and motion effects using the Volterra expansion of the realignment parameters. To isolate an estimate of intrinsic functional connectivity, we regressed voxel-wise BOLD time series against the model incorporating task covariates as nuisance signals and analyzed the residuals of this model. Subsequent to this denoising procedure, the time-series were band-pass filtered (0.009 Hz < *f* < 0.08 Hz). Intrinsic connectivity estimated using this approach has been previously validated with task-free resting state connectivity [32].

To generate whole-brain functional connectomes, we parcellated every individual’s brain image into 333 brain regions or nodes using a high-resolution template based on Gordon et al. [33]. This template uses resting state functional connectivity patterns to define brain parcels that represent putative, functionally coherent, brain areas providing a label based on intrinsic functional brain networks. Intrinsic functional time series were extracted for each of the regions and correlated with every other region to obtain a 333 × 333 inter-regional functional connectivity matrix for every individual. We transformed the correlation coefficients into z-scores using Fisher’s z transformation. The specific choice of a parcellation scheme can impact the results of a network analysis [34–36]. To ensure that our findings are robust irrespective of choice of brain parcellation scheme, we also used a second anatomical parcellation based on the AAL atlas [37] (reported in the supplement).

### Statistical analyses

The statistical analysis was designed in a step-wise manner to address study aims as follows.

#### 1. To identify a connectome-based predicitive biomarker of antidepressant treatment outcome

The Network Based Statistic (NBS) [38] was used to assess differences in pre-treatment functional connectivity between MDD-R and MDD-NR participant groups taken across the three treatment arms. Analogous to cluster-based correction strategies used in voxel-wise MRI studies, the NBS deals with the multiple comparisons problem posed by connectomic data by evaluating the null hypothesis at the level of inter-connected sub-networks rather than individual connections. We first performed a two-sample *t*-test at each connection independently to test for significant differences in the value of connectivity between the two groups. A primary component-forming threshold (*p* < .001) was applied to form a set of supra-threshold connections. Next, the size of the connected components in this thresholded network was computed. In this context, connected components are sets of nodes that can be linked by a set of supra-threshold connections. The statistical significance of the size of each observed component was then evaluated with respect to an empirical null distribution generated by randomly permuting the group membership of each individual, estimating the test statistic on the permuted data, storing the size of the largest component identified in the permuted data, and repeating the analysis (1000 permutations). A corrected *p*-value for each observed component was estimated as the proportion of null component sizes that was larger than the observed value. Observed components with *p* < 0.05, component-wise corrected, were identified as significant sub-networks differentiating the two groups. Functional connectivity estimates for each connection of the identified sub-network were extracted.

In supplementary analyses (Section S2), we tested for associations between connectivity in this connectomic signature with demographic and clinical symptom measures and comparisons between MDD-R & MDD-NR controlling for these measures. We also examined predictive models using this signature in classifying MDD-R/MDD-NR individuals and evaluate additive predictive value relative to demographic and clinical measures in a cross-validation framework (Supplementary Section S3).

For the analyses (2–4) below, we used a single connectivity estimate averaged across the significant connections for the identified network. We also computed an average connectivity estimate for each labeled intrinsic functional network pair combinations that characterized this network for the analyses below. To control for multiple testing due to number of measures, we employed a Benjamini-Hochberg FDR corrected *p* < 0.05 for statistical evaluation.

#### 2. Is the identified connectomic predictive biomarker differentially modulated by different medications?

To test whether functional connectivity in the sub-network identified in analysis 1 is differentially associated with treatment outcome depending on antidepressant type, we used an analysis of variance (ANOVA) with two between-participants factors: treatment outcome (MDD-R/MDD-NR) and antidepressant type (with levels for the SSRIs escitalopram and sertraline, and the serotonin–norepinephrine reuptake inhibitor (SNRI) venlafaxine-XR). We tested for the interaction between outcome and antidepressant treatment type associated with functional connectivity.

#### 3. Does the connectomic predictive biomarker also characterize MDD disease state at baseline?

To test whether functional connectivity in the identified sub-network differs between MDD group as a whole from controls at baseline, we compared extracted connectivity measures using an ANOVA with group (MDD/control) as a between-participants factor. To identify other connectomic diagnostic signatures beyond the identified sub-network, we also performed an exploratory whole-brain connectivity comparison between the MDD and control groups using NBS (Supplementary analyses S4).

#### 4. Does the connectomic predictive biomarker change after 8 weeks of treatment (i.e. is also a response biomarker)?

To test whether functional connectivity of the identified sub-network changed after treatment from baseline, we used an ANOVA with pre vs post follow-up (time) as a within-participants factor and group (with levels for MDD depending on treatment outcome i.e. MDD-R and MDD-NR, and controls) as a between-participants factor. We tested for the interaction between Pre–Post follow-up and group and also performed post hoc tests to characterize any significant interactions.

## Results

Table 1 shows the demographic and clinical characteristics for remitters and non-remitter MDD participants. The remission rate for the sample was 35%.

**Table 1:** Participant demographics and clinical characteristics

	Controls (*n* = 62)	Remitters (*n* = 58)	Non-Remitters (*n* = 105)
%Females	51%	51%	51%
Age (years)	31.4 ± 13.0	29.7 ± 8.7	36.6 ± 12.7
HRSD_17_ baseline		21.7 ± 4.0	21.8 ± 3.6
HRSD_17_ week 8		4.9 ± 1.7	13.3 ± 3.9
HRSD_17_ %change		77.3 ± 8.3	37.8 ± 19.2
Age of onset (years)		19.7 ± 8.1	22.6 ± 10.7
MDD duration (years)		9.5 ± 8.8	13.5 ± 12.2
Treatment naïve (%)		64%	33%
Treatment arm (E/S/V)		19/20/19	39/34/32
%MDD sample		35%	65%

*HRSD*_*17*_ 17-item Hamilton Rating Scale for Depression, *MDD* major depressive disorder, *E* Escitalopram, *S* Sertraline, *V* Venlafaxine-XR

### Pre-treatment network differences in functional connectivity between MDD-R and MDD-NR

**Fig. 1:**
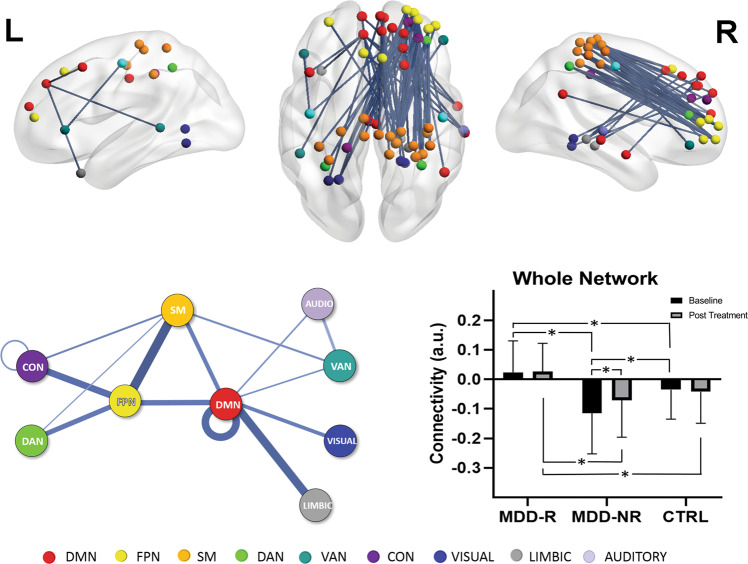
Pre-treatment connectome networks differentiating remitters from non-remitters. (top row) The connectomic feature identified from the NBS analysis. Node colors indicate intrinsic resting state brain network membership as defined by the Gordon et al. [33] parcellation. (bottom row) The patterns of different intra- (loops) and inter-network connections comprising this feature (listed in Table 2) are shown. The thickness of the lines correspond to the number of significant connections between networks of interests relative to the total number of possible connections i.e. thicker lines implies more number of significant connections between the networks. Bar plots (means and SD) showing average connectivity estimates at baseline and post-treatment for each group. There was a significant time*group interaction for average connectivity in this network feature. Asterisks indicate significant post hoc findings (*p* < 0.05) for this interaction. DMN default mode network, FPN fronto-parietal network, SM somatomotor, VAN ventral attention network, DAN dorsal attention network, CON cingulo-opercular network, L left, R right, MDD-R Remitters, MDD-NR Non-remitters, Ctrl Healthy individuals

The NBS analysis identified a connectomic signature comprised of 86 connections across 59 nodes, which was significantly different in pre-treatment functional connectivity between MDD-R and MDD-NR (MDD-R > MDD-NR; *p* = 0.021 corrected for multiple comparisons; Fig. 1 and Table 2). This connectomic signature was characterized by: (1) elevated intra-network intrinsic functional connectivity within the DMN (MDD-R > MDD-NR) and (2) greater inter-network connectivity: (A) between regions of the DMN, fronto-parietal and somatomotor networks; (B) between regions of the DMN and visual, limbic, auditory, and ventral attention networks; and (C) between the fronto-parietal and somatomotor networks with cingulo-opercular and dorsal attention network regions (MDD-R > MDD-NR). Connectivity was associated with age, gender, and treatment naivety, but remained significantly different between groups controlling for these measures. Average connectivity in this signature significantly improved predictive accuracy in classifying remitters from non-remitters compared to a model using demographic and clinical measures alone (*p* < 0.001; Supplementary Table S1: cross-validated accuracy of the model without connectivity/with connectivity = 61.5%/68.8%; sensitivity = 52.6%/63.1%; specificity = 72.4%/72.4%).

**Table 2:** Pre-treatment functional connectivity differences between remitter and non-remitter groups using network-based statistical analysis

Significant connections				Functional connectivity	
Seed region	Coordinates (*x*,*y*,*z*)	Target region	Coordinates (*x*,*y*,*z*)	Remitters (*N* = 58)	Non-remitters (*N* = 105)
				(Mean ± SD)	(Mean ± SD)
*DMN-DMN (7 connections)*					
L - Medial Superior Frontal Gyrus (BA9)	(−5.6, 42.2, 35.1)	L - Middle Frontal Gyrus (BA8)	(−41.7, 16.1, 47.5)	0.797 ± 0.245	0.643 ± 0.268
L - Medial Superior Frontal Gyrus (BA10)	(−6.5, 54.7, 18.1)	R - Mid Cingulum (BA24)	(3, −19.6, 37.9)	0.402 ± 0.273	0.247 ± 0.277
L - Medial Superior Frontal Gyrus (BA9)	(−6.5, 54.7, 18.1)	R - Middle Temporal Gyrus (BA21)	(57.5, −7.4, −16.4)	0.437 ± 0.236	0.266 ± 0.263
R - Medial Superior Frontal Gyrus (BA10)	(8.2, 53.8, 14)	R - Mid Cingulum (BA24)	(3, −19.6, 37.9)	0.418 ± 0.293	0.233 ± 0.318
R - Medial Superior Frontal Gyrus (BA9)	(6.8, 44.5, 34.8)	R - Middle Temporal Gyrus (BA21)	(62.5, −25.6, −5.5)	0.402 ± 0.239	0.252 ± 0.273
R - Superior Frontal Gyrus (BA8)	(13.8, 46.7, 42.1)	R - Middle Temporal Gyrus (BA21)	(57.5, −7.4, −16.4)	0.430 ± 0.226	0.270 ± 0.310
*DMN-FrontoParietal (5 connections)*					
R - Mid Cingulum (BA24)	(3, −19.6, 37.9)	R - Superior Orbitofrontal Gyrus (BA10)	(28.4, 57, −5.1)	0.083 ± 0.160	−0.212 ± 0.231
L - Mid Cingulum (BA24)	(−1.7, −17.7, 39.1)	R - Superior Orbitofrontal Gyrus (BA10)	(28.4, 57, −5.1)	−0.108 ± 0.167	−0.236 ± 0.237
L - Mid Cingulum (BA24)	(−1.7, −17.7, 39.1)	R - Superior Frontal Gyrus (BA10)	(23.5, 59.1, 4.9)	−0.023 ± 0.190	−0.153 ± 0.240
R - Angular Gyrus	(48.9, −53, 28.6)	R - Superior Orbitofrontal Gyrus (BA10)	(28.4, 57, −5.1)	0.476 ± 0.272	0.311 ± 0.303
R - Middle Temporal Gyrus (BA21)	(62.5, −25.6, −5.5)	L - Superior Medial Frontal Gyrus (BA8)	(−5.5, 29.3, 44)	0.355 ± 0.220	0.219 ± 0.239
*DMN-Somatomotor (8 connections)*					
L - Superior Medial Frontal Gyrus (BA9)	(−5.6, 42.2, 35.1)	R - Precentral Gyrus	(19.7, −25, 65.2)	0.007 ± 0.206	−0.127 ± 0.250
R - Superior Medial Frontal Gyrus (BA9)	(6.8, 44.5, 34.8)	R - Precentral Gyrus	(19.7, −25, 65.2)	0.022 ± 0.200	−0.104 ± 0.228
R - Superior Medial Frontal Gyrus (BA9)	(6.8, 44.5, 34.8)	R - Postcentral Gyrus	(11.9, −40.7, 67)	0.087 ± 0.239	−0.055 ± 0.244
R - Superior Medial Frontal Gyrus (BA9/10)	(6.8, 44.5, 34.8)	R - Paracentral Lobule	(4.8, −27.1, 64.8)	0.085 ± 0.223	−0.063 ± 0.272
*DMN-Visual (6 connections)*					
R - Superior Frontal Gyrus (BA8)	(13.8, 46.7, 42.1)	L - Lingual Gyrus (BA18)	(−22, −58.1, 1.5)	0.171 ± 0.231	0.013 ± 0.267
R - Superior Frontal Gyrus (BA8)	(13.8, 46.7, 42.1)	R - Parahippocampal Gyrus (BA19)	(19.6, −45.3, −4.4)	0.106 ± 0.198	−0.020 ± 0.236
R - Medial Superior Frontal Gyrus (BA9)	(5.9, 54.9, 29.4)	L - Fusiform Gyrus	(−28.8, −58.8, −9.1)	−0.011 ± 0.192	−0.145 ± 0.216
R - Medial Superior Frontal Gyrus (BA9)	(5.9, 54.9, 29.4)	R - Lingual Gyrus	(22.3, −46.5, −9.9)	0.067 ± 0.193	−0.062 ± 0.236
*DMN-Limbic (3 connections)*					
R - Superior Frontal Gyrus (BA8)	(21, 32.8, 41.1)	R - Hippocampus	(24.9, −35.9, −4.8)	0.012 ± 0.186	−0.094 ± 0.192
*DMN-Auditory (1 connection)*					
L - Medial Superior Frontal Gyrus (BA9)	(−5.6, 42.2, 35.1)	R - Superior Temporal Gyrus (BA22)	(61.7, −24, 1.3)	0.082 ± 0.188	−0.050 ± 0.247
*DMN-Ventral Attention (1 connection)*					
L - Medial Superior Frontal Gyrus (BA9)	(−5.6, 42.2, 35.1)	L - Middle Temporal Gyrus (BA22)	(−48.1, −40, 2.4)	0.200 ± 0.245	0.043 ± 0.278
*DMN-Unspecified Network (1 connection)*					
L - Medial Superior Frontal Gyrus (BA9)	(−5.6, 42.2, 35.1)	L - Superior Temporal Pole (BA38)	(−33.6, 17.2, −31.5)	0.218 ± 0.214	0.090 ± 0.234
*FrontoParietal-Somatomotor (36 connections)*					
R - Superior Frontal Gyrus (BA10)	(30.9, 52.2, 9.9)	L - Supplementary Motor Area	(−5.4, −15.9, 48.8)	−0.040 ± 0.193	−0.182 ± 0.273
R - Superior Frontal Gyrus (BA10)	(30.9, 52.2, 9.9)	L - Precentral Gyrus	(−20.5, −24.9, 64.5)	−0.122 ± 0.200	−0.248 ± 0.225
R - Superior Frontal Gyrus (BA10)	(30.9, 52.2, 9.9)	R - Precentral Gyrus	(19.7, −25, 65.2)	−0.108 ± 0.224	−0.277 ± 0.200
R - Superior Frontal Gyrus (BA10)	(30.9, 52.2, 9.9)	L - Postcentral Gyrus	(−35.2, −35.3, 42)	0.028 ± 0.203	−0.101 ± 0.225
R - Superior Frontal Gyrus (BA10)	(30.9, 52.2, 9.9)	R - Postcentral Gyrus	(28, −34.8, 63.1)	−0.157 ± 0.241	−0.312 ± 0.237
R - Superior Frontal Gyrus (BA10)	(30.9, 52.2, 9.9)	R - Inferior Parietal Gyrus	(34.2, −40.6, 51.6)	−0.053 ± 0.207	−0.229 ± 0.246
R - Middle Frontal Gyrus	(38.1, 45.9, 7.7)	R - Precentral Gyrus	(19.7, −25, 65.2)	−0.079 ± 0.180	−0.209 ± 0.180
R - Middle Frontal Gyrus	(38.1, 45.9, 7.7)	R - Postcentral Gyrus	(28, −34.8, 63.1)	−0.097 ± 0.224	−0.224 ± 0.204
R - Middle Frontal Gyrus	(38.1, 45.9, 7.7)	R − Inferior Parietal Gyrus	(34.2, −40.6, 51.6)	0.009 ± 0.197	−0.114 ± 0.212
R - Medial Superior Frontal Gyrus (BA8)	(7, 25.7, 47.3)	R - Precentral Gyrus	(16.5, −32.8, 67.7)	−0.003 ± 0.233	−0.128 ± 0.216
R - Medial Superior Frontal Gyrus (BA8)	(7, 25.7, 47.3)	R - Postcentral Gyrus	(11.9, −40.7, 67)	0.100 ± 0.229	−0.034 ± 0.216
R - Medial Superior Frontal Gyrus (BA8)	(7, 25.7, 47.3)	R - Paracentral Lobule	(4.8, −27.1, 64.8)	0.180 ± 0.217	0.046 ± 0.208
R - Superior Orbitofrontal Gyrus (BA10)	(28.4, 57, −5.1)	R - Precentral Gyrus	(16.5, −32.8, 67.7)	−0.100 ± 0.186	−0.226 ± 0.218
R - Superior Orbitofrontal Gyrus (BA10)	(28.4, 57, −5.1)	R - Postcentral Gyrus	(9.5, −42.5, 60.4)	−0.147 ± 0.198	−0.270 ± 0.224
R - Superior Orbitofrontal Gyrus (BA10)	(28.4, 57, −5.1)	L - Paracentral Lobule	(−5, −28.2, 60.4)	−0.017 ± 0.186	−0.163 ± 0.276
R - Superior Orbitofrontal Gyrus (BA10)	(28.4, 57, −5.1)	R - Paracentral Lobule	(4.8, −27.1, 64.8)	0.026 ± 0.222	−0.125 ± 0.265
R - Superior Orbitofrontal Gyrus (BA10)	(28.4, 57, −5.1)	L - Superior Parietal Gyrus (BA5)	(−28.6, −44.7, 61.7)	−0.156 ± 0.179	−0.273 ± 0.196
R - Superior Orbitofrontal Gyrus (BA10)	(28.4, 57, −5.1)	R - Inferior Parietal Gyrus (BA40)	(34.2, −40.6, 51.6)	−0.098 ± 0.186	−0.239 ± 0.219
L - Middle Frontal Gyrus (BA10)	(−28.6, 50.9, 10.1)	R - Precentral Gyrus	(19.7, −25, 65.2)	−0.101 ± 0.196	−0.222 ± 0.216
R - Inferior Frontal Orbitalis	(42.8, 48.3, −5.1)	R - Precentral Gyrus	(19.7, −25, 65.2)	−0.051 ± 0.170	−0.171 ± 0.215
*FrontoParietal-CinguloOpercular (7 connections)*					
R - Superior Frontal Gyrus (BA10)	(30.9, 52.2, 9.9)	L - Mid Cingulum (BA31)	(−16.6, −36.1, 42.7)	−0.127 ± 0.189	−0.258 ± 0.228
R - Superior Frontal Gyrus (BA10)	(30.9, 52.2, 9.9)	R - Mid Cingulum (BA31)	(16.2, −33.1, 43.2)	−0.118 ± 0.193	−0.256 ± 0.215
R - Superior Orbitofrontal Gyrus (BA10)	(28.4, 57, −5.1)	L - Mid Cingulum (BA31)	(−16.6, −36.1, 42.7)	−0.155 ± 0.175	−0.300 ± 0.211
R - Superior Orbitofrontal Gyrus (BA10)	(28.4, 57, −5.1)	R - Mid Cingulum (BA31)	(16.2, −33.1, 43.2)	−0.144 ± 0.175	−0.299 ± 0.211
R - Middle Frontal Gyrus	(38.1, 45.9, 7.7)	R - Mid Cingulum (BA31)	(16.2, −33.1, 43.2)	−0.003 ± 0.230	−0.132 ± 0.203
*FrontoParietal-Dorsal Attention (4 connections)*					
R - Superior Frontal Gyrus (BA10)	(30.9, 52.2, 9.9)	R - Inferior Parietal Gyrus (BA40)	(33.5, −48.2, 49.4)	−0.081 ± 0.181	−0.238 ± 0.293
R - Superior Orbitofrontal Gyrus (BA10)	(28.4, 57, −5.1)	R - Inferior Parietal Gyrus (BA40)	(33.5, −48.2, 49.4)	−0.003 ± 0.195	−0.184 ± 0.287
R - Superior Orbitofrontal Gyrus (BA10)	(28.4, 57, −5.1)	L - Inferior Parietal Gyrus (BA40)	(−31.1, −48.9, 47.1)	−0.085 ± 0.187	−0.217 ± 0.247
*Somatomotor-CinguloOpercular (3 connections)*					
R - Precentral Gyrus	(19.7, −25, 65.2)	R - Middle Frontal Gyrus (BA10)	(31.3, 39.7, 25.6)	−0.076 ± 0.197	−0.204 ± 0.211
*Somatomotor-Ventral Attention (2 connections)*					
L - Postcentral Gyrus	(−41.5, −12.5 50.4)	L - Inferior Frontal Triangularis (BA47)	(−45.4, 28.8, 0.8)	0.150 ± 0.222	0.029 ± 0.205
R - Paracentral Lobule	(4.8, −27.1, 64.8)	R - Inferior Frontal Orbitalis (BA47)	(48.1, 38.3, −9.2)	0.160 ± 0.158	0.024 ± 0.263
*Somatomotor-Dorsal Attention (1 connection)*					
R - Precentral Gyrus	(19.7, −25, 65.2)	R - Middle Frontal Gyrus (BA10)	(36.8, 37.8, 13.1)	−0.070 ± 0.185	−0.192 ± 0.213
*CinguloOpercular–CinguloOpercular (1 connection)*					
R - Middle Frontal Gyrus (BA10)	(31.3, 39.7, 25.6)	R - Mid Cingulum (BA31)	(16.2, −33.1, 43.2)	0.150 ± 0.295	−0.004 ± 0.249

The seed and target regions for the significant connections from this identified connectomic network were defined as belonging to the intrinsic functional brain networks based on the established parcellation template of Gordon et al. [33] rather than by anatomical boundaries (which means that an anatomical region could have membership for multiple intrinsic functional network due to multiple nodes representing the region). Fifteen unique network-pair combinations were identified and are listed. Mean and standard deviations for the resting functional connectivity estimates for the different connections are summarized

### Is this connectomic predictive biomarker differentially associated with outcome depending on type of antidepressant?

There were no significant interactions between treatment outcome and type of antidepressant or main effect of antidepressant for connectivity measures for this signature, suggesting this biomarker to be associated with a general response to the three antidepressant medications.

### Does the connectomic predictive biomarker also characterize MDD disease state at baseline?

At baseline, controls were not significantly different compared to the whole MDD cohort for average connectivity in this signature (*p* = 0.078). However, controls had significantly greater connectivity compared to MDD only in the DMN-frontoparietal connections of this signature (FDRp < 0.05).

### Does this connectomic predictive biomarker change after 8-week antidepressant treatment?

**Fig. 2:**
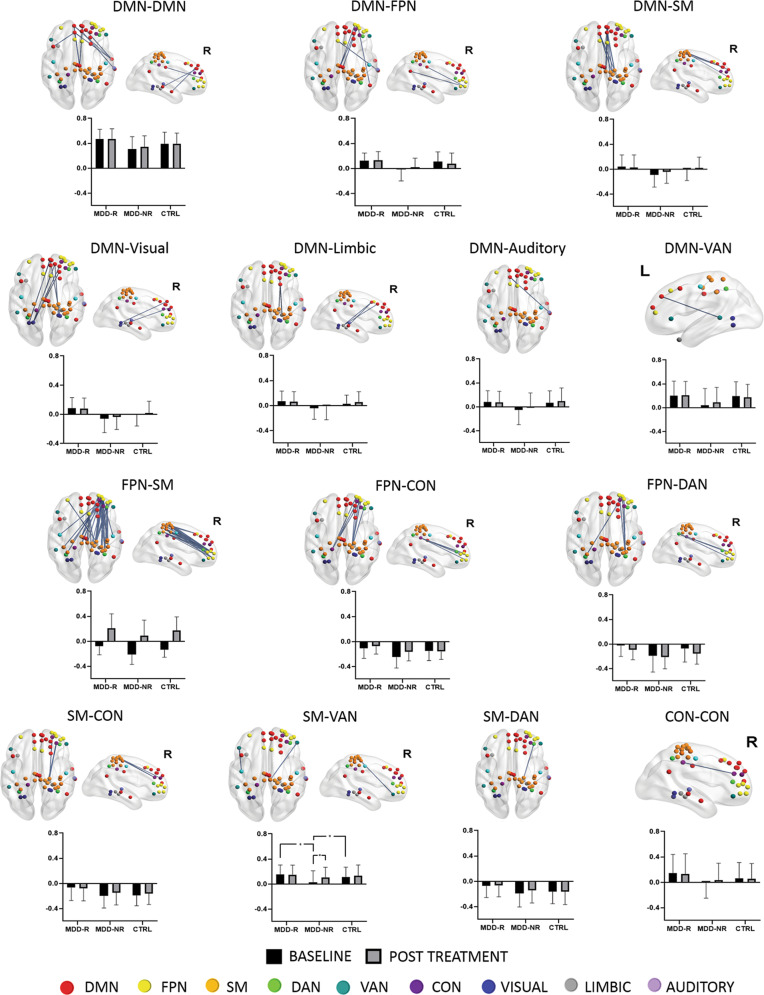
Intra- and inter-network connections that differentiate Remitters from Non-Remitters. Bar plots show connectivity estimates at baseline and post-treatment (means and SD). There was a significant time*group interaction (FDRp < 0.05) only for the somatomotor-ventral attention connections. Asterisks indicate significant post hoc findings (*p* < 0.05) for this interaction. DMN default mode network, FPN fronto-parietal network, SM somatomotor, VAN ventral attention network, DAN dorsal attention network, CON cingulo-opercular network, L left, R right, MDD-R Remitters, MDD-NR Non-Remitters, Ctrl Healthy individuals

A significant group*time interaction was observed for average connectivity in this signature (*p* = 0.011) and specifically only for connections between the somatomotor and ventral attention networks (FDRp < 0.05; Table 3 and Fig. 2). Post hoc contrasts indicated a significant increase of functional connectivity with treatment only for MDD-NR (controls and MDD-R remain unchanged). At baseline, MDD-NR had significantly lower connectivity relative to both MDD-R and controls. Post-treatment, although connectivity had normalized (i.e., there was no significant difference relative to controls), MDD-NR still had a significantly lower average connectivity in the signature than MDD-R. On the other hand, MDD-R had a significantly higher average connectivity in this signature relative to controls at both pre- and post-treatment. For the somatomotor-ventral attention connectivity, MDD-R were not significantly different than controls at both time-points and also relative to MDD-NR at post-treatment.

**Table 3:** Change in functional connectivity following 8 weeks of antidepressant treatment

Networks	Functional connectivity (Mean ± SD)						*p* values									
	Ctrl		MDD-R		MDD-NR		Time*Group	Main effect time (Baseline vs Post)			Baseline only—between group (age controlled)			Post Tx only—between group (age controlled)		
	Baseline	Post	Baseline	Post	Baseline	Post		MDD-R	MDD-NR	Ctrl	MDD-R > MDD-NR	MDD-NR<Ctrl	MDD-R > Ctrl	MDD-R > MDD-NR	MDD-NR<Ctrl	MDD-R > Ctrl
Whole network	−0.039 ± 0.099	−0.042 ± 0.106	0.034 ± 0.103	0.026 ± 0.096	−0.117 ± 0.142	−0.071 ± 0.125	0.011	NS	0.003	NS	<0.001	0.001	0.011	<0.001	NS	0.002
DMN-FrontoParietal	0.107 ± 0.158	0.079 ± 0.166	0.121 ± 0.125	0.130 ± 0.140	−0.014 ± 0.186	0.020 ± 0.144	0.031	NS	0.045	NS	<0.001	<.001	NS	<0.001	NS	0.049
DMN-Somatomotor	−0.010 ± 0.177	0.016 ± 0.178	0.052 ± 0.188	0.028 ± 0.201	−0.099 ± 0.196	−0.044 ± 0.182	0.014	NS	0.002	NS	0.001	0.022	NS	NS	NS	NS
FrontoParietal-Somatomotor	−0.141 ± 0.117	−0.158 ± 0.128	−0.064 ± 0.138	−0.071 ± 0.127	−0.211 ± 0.163	−0.164 ± 0.145	0.016	NS	0.008	NS	<0.001	0.009	0.024	0.001	NS	0.001
FrontoParietal-Dorsal Attention	−0.077 ± 0.223	−0.112 ± 0.185	−0.016 ± 0.176	−0.019 ± 0.165	−0.194 ± 0.271	−0.134 ± 0.217	0.015	NS	0.019	NS	<0.001	0.008	NS	0.007	NS	0.018
Somatomotor-Ventral Attention	0.105 ± 0.154	0.129 ± 0.176	0.156 ± 0.156	0.146 ± 0.156	0.027 ± 0.187	0.101 ± 0.167	0.003*	NS	<0.001	NS	0.001	0.034	NS	NS	NS	NS

Network connections with a significant Time*Group interaction are listed. (*Significant at FDR corrected *p* < 0.05)

## Discussion

Using a comprehensive, connectome-wide analysis, we examined pre-treatment intrinsic functional connectivity associated with remission after 8 weeks of treatment with one of three commonly prescribed antidepressants: two selective serotonin reuptake inhibitors (escitalopram and sertraline) and a combined serotonin and norepinephrine reuptake inhibitor (venlafaxine-XR).

Our study focuses on large-scale intrinsic brain networks that play a key role in diverse cognitive, emotional, and self-reflective functions [7] and sheds light on network-level connectivity in a sample powered to stratify by remission status and different antidepressant types. Patients with an overall greater than normal connectivity, particularly related to the DMN, fronto-parietal and somatomotor brain networks, were the most likely to benefit from antidepressant treatment and to achieve acute remission. This finding was especially striking given that, as a total group, depressed patients were characterized by a connectomic signature of lower connectivity compared to controls at the pre-treatment baseline (Supplementary Fig. S2 and Table S2). This finding was robust to the method used to define these networks (Supplementary Fig. S3 and Table S3). The new insights from this analysis suggest that clinical remission may in fact require intact or greater than normal pre-treatment inter-network intrinsic connectivity, rather than reflect a shift from abnormal to normal connectivity. This effect appears especially salient for brain networks associated with awareness of self and cognitive control.

The DMN is the network of the brain that focuses on internal mental states and its activity is often anti-correlated with other intrinsic networks involved in attending to functions such as attentional vigilance and orienting [39, 40]. This interplay between the DMN with other brain networks and its relation to antidepressant treatment response has been of interest in previous work using electroencephalography. Using a LORETA analysis with a region of interest approach, Whitton and colleagues [41] evaluated whether activation of the rostral anterior cingulate cortex (rACC) shows phase lagged synchronization with other cortical regions in the beta and theta frequency bands. They found that synchrony of the rACC and anterior insula within the theta band is a prospective non-specific marker of response to both sertraline and placebo. Our study complements and builds on this knowledge in several ways. We use a different methodology based on functional MRI applied in a connectome-wide approach to evaluate intrinsic functional connectivity across networks. We deploy this methodology in a biomarker trial comparing sertraline to two other active antidepressant treatments. In addition, we evaluate change in functional connectivity post-treatment and compared this change to a normative framework of healthy subject connectivity over the same time period. Our findings indicate that connectivity related to the DMN is a prognostic marker of remission across all three treatments. Thus, our findings add weight to the possibility that connectivity or synchronization, assessed both regionally and brain-wide, and with different methodologies, may be an important general marker of antidepressant treatment outcomes. Our findings also support previous reports of functional and structural connectivity of the DMN to be associated with antidepressant treatment outcomes in depression [10, 11, 42–44]. Non-remitters in our study showed abnormally reduced average functional connectivity in our connectomic signature. This is consistent with previous findings that also observed similar low pre-treatment functional connectivity, though restricted to the cognitive control network, to be associated with non-remission to escitalopram in older depressed cohorts [16]. Our findings however contradict previous reports that treatment resistant patients show hyper-connectivity within the DMN compared to those who respond to treatment [45]. However, previous studies have typically measured response (i.e. symptom improvement), rather than remission and have relied on limited sample sizes. While response may reflect an immediate relief of symptoms, achieving remission is the ultimate goal of treatment. This inconsistency in direction of findings could also possibly be because previous studies have utilized resting state fMRI whereas our study derived intrinsic connectivity from task-based fMRI scans after removing task residuals. These differences may be related to task effects not fully captured by the task regressors, or more simply differences in pre-processing strategies implemented by different studies [46]. It is likely that the observed increases in connectivity with the DMN may represent diminished task-induced deactivation of this network, and vice versa, which would suggest that the group differences could be related to differences in attentional engagement rather than solely due to internal rumination.

Our data also provide insight into the impact of medications on neural connectivity. Previous studies have observed an increase [47], decrease [11, 17] or both [48] in resting functional connectivity due to antidepressant medications. Our MDD cohort overall had a significant increase in connectivity (averaged over the identified predictive connectomic signature) from baseline with normalization at post-treatment. However, on splitting the group based on outcome, only non-remitters showed an increase in connectivity at the follow-up scan. While this was reflected in average connectivity across the predictive connectomic signature, this effect was found strongest for connectivity between the inferior frontal regions of the VAN and post-central gyral regions of the somatomotor network. Depression is characterized by deficits in reorienting attention to salient events and these results suggest the role of antidepressants in possibly targeting connections relevant in relaying information about salience and somatosensation [8]. For these patients who did not remit in the acute phase of focus in this study, there was also a lack of correlation between change in connectivity and change in symptoms, consistent with their non-remitting status (supplementary results). It remains possible that these participants are on a trajectory to remit over a longer time scale (subsequent to the increase in connectivity), which requires future investigation. Remitters (and controls), on the other hand, exhibited consistent connectivity across baseline and follow-up scans. This observation is important for developing a mechanistic understanding of how connectivity relates to clinical outcome. In this case, it appears that “acute” remission depends on the pre-existence of higher than normal levels of intrinsic network organization, particularly for regions critical in top-down control of attention and emotions which are often found to be underlying impaired cognitive and emotion processing abilities in depression [29, 49]. Without this, the brain may be unable to mount the plastic change required for clinical change. Although we would presume that any clinical change (symptom improvement or remission) would necessitate an underlying change at the neural level, this may not be true for intrinsic functional connectivity. It is possible that task-elicited neural changes are more directly related to the clinical change over and above the intact underlying intrinsic organization of what is occurring at rest [50]. However, this possibility requires systematic investigation, particularly as there is a dearth of studies that have evaluated how changes in resting connectivity relate to improvement in symptoms following antidepressant medication treatments. One reason for discrepant results may be the use of task residuals in our study, although there is strong similarity between resting and residual brain networks [32, 51]. Future studies should also evaluate differential contributions of resting and task-related functional connectivity.

Our study also provides evidence on how intrinsic connectivity relates to outcome from different types of antidepressants. To the best of our knowledge, no previous study has compared effect of different types of antidepressants on intrinsic functional connectivity measures. In our study, MDD participants were randomized to one of three commonly used antidepressants in primary care settings. The connectomic signature identified in our study appears to be associated with a general response to antidepressant medications and we did not observe connectivity differences associated with the three antidepressants used. This could suggest that greater than normal intrinsic organization is likely a necessary moderator of broad types of drug treatments of depression. Although this remains to be tested in medications beyond the ones used in our study. Clinically this could mean that MDD patients with an abnormally reduced intrinsic connectivity are less likely to benefit acutely from antidepressant medications as first-line treatments. Whether these patients could benefit from an alternative treatment regime that normalizes this DMN connectivity prior to or in conjunction with antidepressants is worthy of future investigations.

Our findings are limited to the three commonly prescribed antidepressant medications used in the study, and the generalizability of these findings to other classes of antidepressants currently available needs to be validated. Our study also lacked a placebo arm, which limits our ability to differentiate the specific effects of antidepressant therapy from spontaneous remission. Previous findings have identified a role for resting-state connectivity in predicting placebo effects [15]. We also included participants who were medication naïve or had previous history of antidepressant use. Although our network findings were significant, even after controlling for previous treatment in our analysis and patients on existing treatments underwent a washout phase prior to enrolling in the current study, any bias due to effects of previous treatments on current antidepressant action cannot be ruled out. We performed cross-validation classification analyses to predict treatment outcome mainly to identify the best predictive model features and provide an operational example of how neural measures we identified could be helpful in treatment decision. Despite our respectable sample size relative to prior studies investigating neural predictors of antidepressant treatment and we used cross-validation statistics, these findings can only be considered preliminary without replication in an independent cohort. Integration of both task-evoked and intrinsic functional connectivity will be an interesting avenue of future investigation [52, 53].

The use of connectomics to identify novel brain networks in diagnosis and prognosis of psychiatric disorders is a major methodological step forward. Using this approach, we have identified the intrinsic brain networks underlying acute non-remission to antidepressants. Greater than normal connectivity within these networks may be a prerequisite mechanism for recovery on antidepressant medications. This identified connectomic signature holds potential as a prognostic marker in the clinical management of depression.

## Supplementary information

Supplementary Section
