# Supplementary material for: Intrinsic connectomes are a predictive biomarker of remission in major depressive disorder
Source: Mol Psychiatry. 2019 Nov 6;25(7):1537–49. doi: 10.1038/s41380-019-0574-2 (PMC7303006; doi:10.1038/s41380-019-0574-2)
Supplement: Supplementary file 1 — Supplementary Section [file 41380_2019_574_MOESM1_ESM.docx]

**Supplementary Section**

**Intrinsic connectomes are a predictive biomarker of remission in major depressive disorder.**

Mayuresh S. Korgaonkar, PhD ^a,b^, Andrea N. Goldstein-Piekarski, PhD^c,d^, Alexander Fornito, PhD^e^, Leanne M. Williams, PhD^a,c,d^

^a^ The Brain Dynamics Centre, Westmead Institute for Medical Research, The University of Sydney, Sydney, Australia

^b^ Discipline of Psychiatry, Western Clinical School, The University of Sydney, Sydney, Australia

^c^ Department of Psychiatry and Behavioral Sciences, Stanford University, Stanford, California, USA

^d^ Sierra-Pacific Mental Illness Research, Education, and Clinical Center (MIRECC) VA Palo Alto Health Care System, Palo Alto, CA, USA

^e^ Brain and Mental Health Research Hub, Monash Institute of Cognitive and Clinical Neurosciences & Monash Biomedical Imaging, Monash University, Clayton, Victoria, Australia

Supplementary Figure 1: CONSORT chart

S1: fMRI acquisition and preprocessing methods

S2: Associations of connectivity with demographic and clinical measures

S3: Predictive models using the identified connectomic signature in classifying remitters from non-remitters pretreatment

S4: NBS analyses comparing whole MDD group with Controls

S5: Replication of main connectome analyses using the AAL parcellation scheme

**Supplementary figure 1: CONSORT CHART**


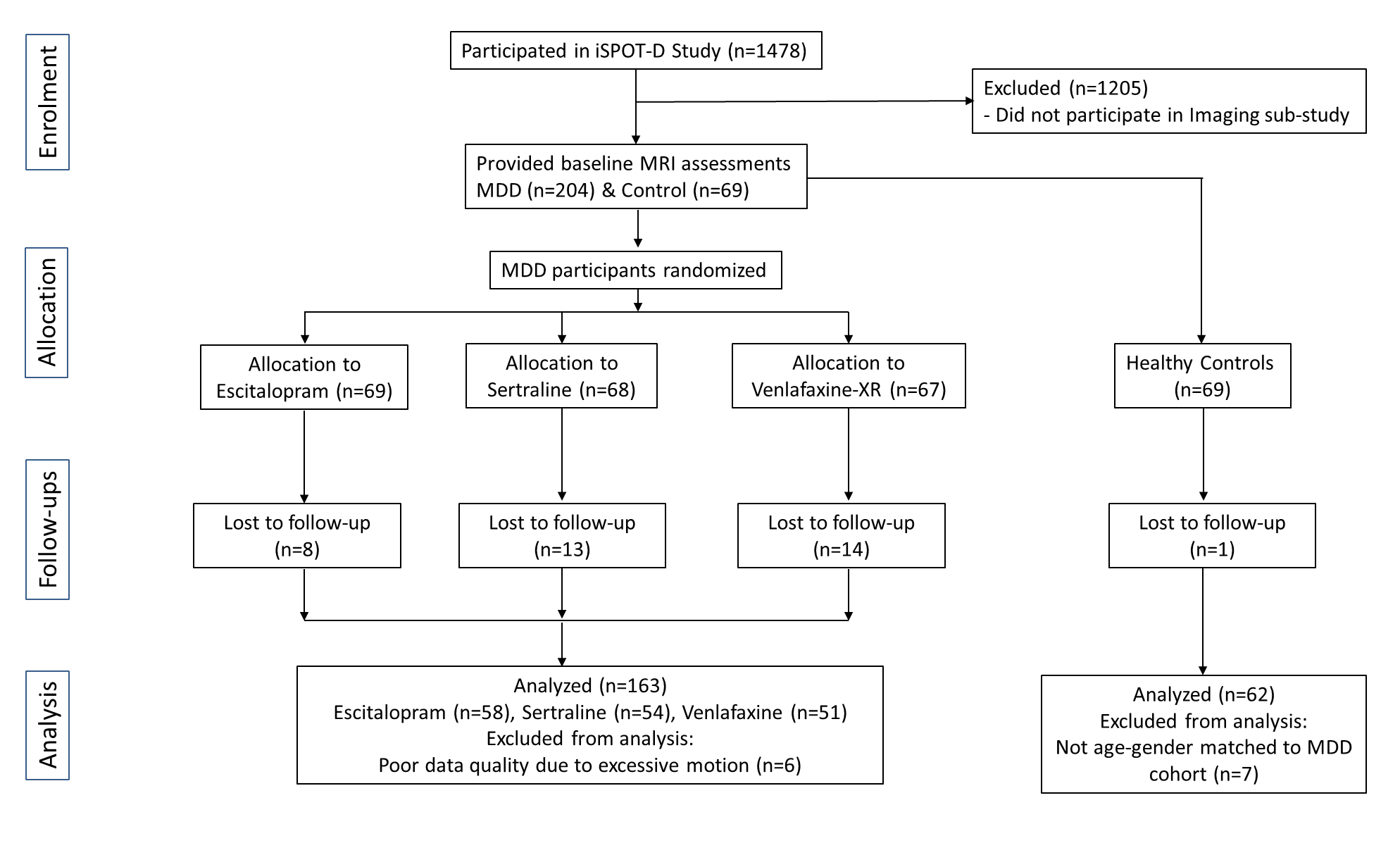


**S1: fMRI acquisition and preprocessing methods**

MRI data for both visits was acquired on a 3T GE Signa HDx scanner (GE Healthcare, Wisconsin) using an 8-channel head coil. MRI acquisition included five fMRI tasks and a 3D T1-weighted structural MRI scan (TR/TE=8.3/3.2ms, Flip Angle=11°, TI=500ms, 256x256 matrix, 180 sagittal 1mm slices, 1mm isotropic voxels). MR images for each task were acquired using echo planar imaging (TR/TE=2500/27.5ms, Flip Angle=90°, 64x64 matrix, FOV=24cm, 40 axial 3.5mm slices covering the whole brain, 120 volumes). The details of the five tasks have been previously described ([1](#_ENREF_1)). Briefly, tasks assessed (a) selective attention using an auditory oddball task, (b) working memory using a continuous performance task, (c) inhibition processes using a Go-NoGo task, and (d) conscious and (e) non-conscious processing of emotional faces. Intrinsic functional connectivity was estimated using data from all five tasks as described below.

***Preprocessing of Connectivity Data***

Preprocessing and data analysis were performed using Statistical Parametric Mapping software (SPM8; Wellcome Department of Cognitive Neurology, London). First images were motion-corrected and corrected for geometric distortions using realignment and unwarping, and then quality control diagnostics were completed on the time series data for each run. Data volumes that were associated with high 1) movement (framewise displacement from one time point to the next) or 2) changes in BOLD signal intensity (as indexed by spatial standard deviation of successive difference images [DVARS] ([2](#_ENREF_2))) were censored (temporally masked) to reduce the influence of motion and related artifacts. Framewise displacement was calculated as the sum of the absolute values of the differentiated realignment estimates as in Power et al., 2014 ([3](#_ENREF_3)). Volumes were censored using established thresholds of framewise displacement greater than or equal to 0.3mm and scaled signal intensity differences greater than 10) ([2-4](#_ENREF_2)). Censoring was implemented with the time series difference analysis toolbox http://www.fil.ion.ucl.ac.uk/spm/ext/#TSDiffAna and in house scripts. A temporal mask was then created for each censored volume and used as regressors of no interest in the first level statistical models ([2](#_ENREF_2), [3](#_ENREF_3)). Since movement related artifacts have been shown to impact volumes acquired before and several seconds after a movement spike, to reduce the influence of movement related artifacts a total of four temporal masks were created for each movement spike (an additional volume before and 2 volumes after the movement spike) ([3](#_ENREF_3)). For signal change spikes, the temporal mask included the censored volume as well as one subsequent volume. Images were then slice-time corrected in SPM8. Following slice time correction, images were spatially normalized to the stereotactic Montreal Neurological Institute (MNI) space using the FMRIB nonlinear registration tool ([5](#_ENREF_5)) using the T1 structural data and smoothed using an 8 mm full-width-at-half-maximum Gaussian kernel in SPM8.

For each fMRI task, the BOLD responses for each experimental condition were modeled in the general linear model framework: oddball (target and non-target trials), continuous performance (working memory, 1-back and baseline trials), Go-NoGo tasks (Go and NoGo trials), and both emotion tasks (each emotion type). Motion effects were also modeled for each task using the Volterra expansion of the realignment parameters proposed in ([6](#_ENREF_6)) (24 regressors; R_t_, R_t_^2^, R_t-1_, R_t-1_^2^ where R’s are the realignment parameters estimated during the preprocessing stage). Additional covariates for each task included the mean signal time course extracted from eroded ventricle and white matter masks, as well as the temporal masks derived from the volume censoring described above. To isolate an estimate of intrinsic functional connectivity, we regressed voxelwise BOLD time series against the model incorporating task covariates as nuisance signals and analysed the residuals of this model. Subsequent to this denoising procedure, the timeseries were band-pass filtered (0.009 Hz < f < 0.08 Hz). Intrinsic connectivity estimated using this approach has been previously validated with task-free resting state connectivity ([7](#_ENREF_7)).

**S2: Associations of connectivity with demographic and clinical measures**

We performed a multivariate ANOVA analysis for each demographic and clinical measure to test association with functional connectivity measures for the whole MDD group. For demographic and clinical measures that were significantly associated with connectivity measures, we tested if connectivity differences between MDD-R and MDD-NR continued to remain significant after controlling for these demographic and clinical measures. As done in the main text, we tested associations with 1) a single connectivity estimate averaged across the significant links for the identified network; and 2) average connectivity estimate for the 15 intrinsic functional network pair combinations that characterised this network (listed in Table 2).

***Results:***

For the whole MDD group, age, gender, duration of MDD, melancholia and treatment naivety were significantly associated with functional connectivity within this signature (all p<0.05). Baseline symptom severity (HRSD_17_), DASS measures, age of onset or number of previous episodes were not found associated with any of the connectivity measures.

Age (p<0.001) and duration of MDD (p<0.005) were negatively associated with connectivity. Connectivity was reduced for females compared to males (p=0.006), those who were not melancholic (p=0.030) and for those who underwent previous MDD treatment (p=0.008). Controlling for age, gender, duration of MDD and treatment naivety, remitters were still significantly different from non-remitters for connectivity averaged across the identified network (p<0.001); and for 13 of the 15 network pairs (all FDR p<0.05 except for DMN-auditory and DMN-ventral attention connections which were no longer significant) suggesting that the connectivity differences between remitters and non-remitters existed beyond demographic and clinical measures.

***Additional correlations of baseline depression severity with functional connectivity in MDD-R***

Considering that remitters had increased connectivity than non-remitters at pre-treatment, we evaluated if baseline connectivity in the predictive network was associated with baseline symptom severity (HRSD_17_) in remitters. There were no significant associations for either average connectivity or any of the specific network links with symptom severity in remitters.

***Change in functional connectivity associated with change in depressive symptoms in MDD-NR***

We evaluated if the increases in connectivity seen with treatment in the non-remitter group is also correlated with symptom reductions (i.e. in HRSD_17_). Average connectivity in our identified connectome network was not associated with symptom change; however, greater increase in connectivity for the DMN-Ventral Attention (p=0.035) and FrontoParietal-CinguloOpercular (p=0.043) networks were found associated with worsening of symptoms. These effects however did not remain significant after the FDR correction.

**S3: Predictive models using the identified connectomic signature in classifying remitters from non-remitters pretreatment**

We examined the predictive performance of the identified connectomic signature in classifying MDD-R from MDD-NR prior to treatment using cross-validation analyses. To illustrate how the functional markers we identified could be helpful in a treatment decision and to inform future studies, we first evaluated the predictive performance using demographic and clinical measures and whether this was improved by using connectivity estimates for our identified connectomic signature. We first then identified the demographic and clinical features that distinguished remitters from non-remitters and the significant variables were included in the first block of a binary regression. In the same model, as block 2, the network connectivity measures (in Table 2) were entered in a backward stepwise (Wald) method, to determine which variables contributed significantly to the model, and whether they improved the model’s prediction of remission above and beyond clinical variables. We ran two models for block 2: 1) using the average connectivity measures for the connectomic signature; and 2) using connectivity values for the individual network pairs that comprised of the connectomic signature. This was mainly done to identify the most predictive features within the connectomic signature.

Cross-validation analyses were then run for both models (first a clinical variable only model in block 1, followed by the combined model in block 2). The dataset was randomly split into an approximately 70% training dataset and 30% testing dataset. The training dataset was then bootstrapped two hundred times, and binary logistic regressions were performed on each bootstrapped dataset. Model coefficients from each predictor were extracted and averaged across all results. These averaged coefficients were then used in a binary logistic regression model on the test data to determine the model’s cross-validated accuracy statistics for classifying MDD-R vs. MDD-NR. Accuracy statistics (sensitivity, specificity, positive predictive values, and negative predictive values) are reported for the models as well as those that were derived from cross-validation.

***Results:***

Age (p<0.001), previous treatment (p<0.001), duration of MDD (p=0.002), all significantly differed between MDD-R and MDD-NR groups (all MDD-NR>MDD-R) and were used as features for the block 1 clinical variables only model. There were no significant differences for other variables (gender, baseline HRSD_17_, age of onset, melancholic, number of episodes or anxiety comorbidity). For block 2, the neural measures that were retained at the end of the stepwise backward regression were: DMN–Visual and FrontoParietal–Somatomotor connectivity. The combined models were significantly improved as compared to the clinical only block 1 model (p<0.001). Cross-validated model accuracies are reported in Supplementary Table S1.

**Supplementary Table S1: Predictive Models using demographic/clinical measures alone and combined models using connectivity measures for the identified connectomic signatures. Both combined models significantly improved predictive accuracies as compared to the clinical only model to classify prospective remitters from non-remitters.**

|  | **Variable** | **Coefficient β values** | **% accuracy**  **(95% CI)** | **% specificity** | **%**  **sensitivity** | **Positive/Negative Predictive Value** |
| --- | --- | --- | --- | --- | --- | --- |
| ***Clinical only model*** | | | | | |  |
| *Training model* |  |  | 72.8%  (64-81%) | 48.7% | 85.3% | 76.2%/63.3% |
| *Cross-validated model* |  |  | 61.5%  (49-78%) | 52.6% | 72.4% | 70.0%/55.5% |
|  | Age | -0.07 |  |  |  |  |
|  | Previous Treatment | -1.17 |  |  |  |  |
|  | MDD Duration | -0.007 |  |  |  |  |
|  |  |  |  |  |  |  |
| ***Combined Model 1*** | | | | | |  |
| *Training model* |  |  | 80.7%  (72-87%) | 61.5% | 90.6% | 81.9%/77.4% |
| *Cross-validated model* |  |  | 68.8%  (54-81%) | 57.8% | 75.8% | 73.3%/61.1% |
|  | Age | -0.03 |  |  |  |  |
|  | Previous Treatment | -1.20 |  |  |  |  |
|  | MDD Duration | -0.02 |  |  |  |  |
|  | Whole network connectivity | 14.43 |  |  |  |  |
|  | | | | | |  |
|  | | | | | |  |
| ***Combined Model 2*** | | | | | |  |
| *Training model* |  |  | 81.5%  (73.2-88.2%) | 61.5% | 92.0% | 82.1%/80.0% |
| *Cross-validated model* |  |  | 68.8%  (54-81%) | 63.1% | 72.4% | 75%/60.0% |
|  | Age | -0.07 |  |  |  |  |
|  | Previous Treatment | -1.48 |  |  |  |  |
|  | MDD Duration | -0.0003 |  |  |  |  |
|  | DMN-Visual | 5.76 |  |  |  |  |
|  | FrontoParietal-Somatomotor | 8.47 |  |  |  |  |
|  |  |  |  |  |  |  |

**S4: Network Based Statistical analyses comparing whole MDD group with Controls**

Whole brain connectivity comparisons between the entire MDD group and Controls (matched for age and gender) were conducted using NBS analyses at baseline. As described in the main text, a two-sample t-test at each edge independently to test for significant differences in the value of connectivity between the two groups was first performed at p<0.001 to identify components or sub-networks and then the statistical significance of the size of each observed component was then evaluated with respect to an empirical null distribution at a p<0.05 corrected for multiple comparisons. This analysis identified a connectomic signature comprised of 137 edges connecting 98 nodes, which was significantly different in functional connectivity between the MDD and control groups (corrected p=0.041) with lower connectivity in the MDD group relative to controls (Supplementary Figure S2; Supplementary Table S2).

***Change in connectivity post treatment i.e. to evaluate whether this connectomic diagnostic biomarker also a response biomarker of antidepressant treatment***

Next, we extracted connectivity estimates in this MDD-Control network and assessed changes between MDD and Controls at post-treatment and also change in functional connectivity from baseline to post treatment in the MDD group. As done in the main text, we estimated: 1) a single connectivity estimate averaged across the significant links for the identified network; and 2) average connectivity estimate for the 23 intrinsic functional network pair combinations that characterised this network. Average connectivity in this network was unchanged from baseline to post treatment in the MDD group and also continued to remain significantly lower relative to controls after treatment. Only the fronto-parietal and somatomotor connectivity significantly increased post-treatment in the MDD group (FDR p<0.05).

***If pre-treatment connectivity in this network could differentiate MDD-R and MDD-NR i.e. to evaluate whether this connectomic diagnostic biomarker also a predictive biomarker of antidepressant treatment***

We also tested if pre-treatment connectivity in this MDD-Control network could differentiate remitters and non-remitter MDD patients. Average connectivity in this network was not significantly different between MDD-R and MDD-NR groups at baseline. Specifically, only the DMN-frontoparietal connectivity was different between MDD-R and MDD-NR groups (FDR p<0.05), a finding which was also observed in the NBS analysis comparing the MDD-R and MDD-NR groups.

***If pre-treatment connectivity in this network is associated with depression severity at baseline***

In the whole MDD cohort, average connectivity in the identified MDD vs. Control network was not associated with depression severity; however, greater connectivity within the CinguloOpercular-Salience (p=0.003) and Salience-Dorsal Attention (p=0.018) networks was associated with lower symptoms. These effects did not remain significant after FDR correction.

**
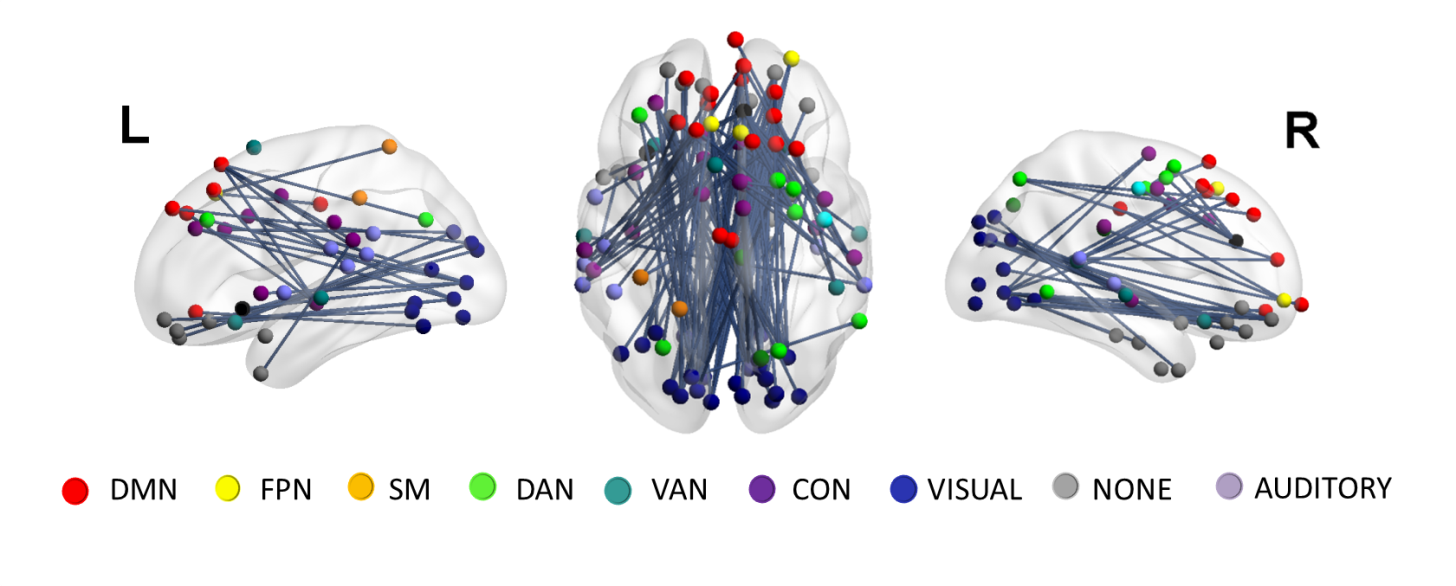
Supplementary Figure S2: Significant connectivity differences between MDD and controls from the NBS analysis (MDD<Controls).** Colors of the nodes represent the intrinsic brain networks that they belong to. *Abbreviations: DMN, default mode network; FPN, fronto-parietal network; SM, somatomotor; VAN, ventral attention network; DAN, dorsal attention network; CON, cingulo-opercular network; L, left; R, right*

**Supplementary Table S2: Pretreatment functional connectivity differences between MDD and control groups using network-based statistical analysis.** Mean and standard deviations for the resting functional connectivity estimates for the different intrinsic network pairs at baseline and post treatment are summarized. P values indicate differences between MDD and Control groups at baseline and post treatment and also change in connectivity from baseline to post treatment for the MDD group. *significant at FDR p<0.05

| **Networks** | Functional Connectivity | | | | p values | | |
| --- | --- | --- | --- | --- | --- | --- | --- |
|  | Ctrl  (n=62) |  | MDD  (n=163) | | MDD vs Ctrl | MDD vs Ctrl | MDD change over time (T1 vs T2) |
|  |  |  |  |  | at T1 | at T2 |  |
|  | Baseline | Post | Baseline | Post |  |  |  |
| Whole network | 0.053 ± 0.071 | 0.002 ± 0.062 | -0.056 ± 0.081 | -0.046 ± 0.086 | <0.001 | <0.001 | NS |
|  |  |  |  |  |  |  |  |
| DMN-FrontoParietal | 0.125 ± 0.161 | 0.082 ± 0.167 | 0.017 ± 0.190 | 0.048 ± 0.185 | <0.001 | NS | 0.019 |
| DMN-Auditory | -0.048 ± 0.139 | -0.105 ± 0.146 | -0.165 ± 0.158 | -0.154 ± 0.177 | <0.001 | 0.016* | NS |
| DMN-CinguloOpercular | -0.064 ± 0.137 | -0.087 ± 0.135 | -0.165 ± 0.138 | -0.170 ± 0.149 | <0.001 | <0.001* | NS |
| DMN-Dorsal Attention | -0.075 ± 0.150 | -0.081 ± 0.130 | -0.189 ± 0.171 | -0.152 ± 0.182 | <0.001 | 0.005* | 0.011 |
| DMN-Ventral Attention | 0.001 ± 0.202 | -0.072 ± 0.236 | -0.101 ± 0.215 | -0.101 ± 0.217 | <0.001 | NS | NS |
| DMN-Visual | 0.088 ± 0.123 | -0.000 ± 0.130 | -0.017 ± 0.148 | -0.013 ± 0.132 | <0.001 | NS | NS |
| Visual-FrontoParietal | -0.019 ± 0.192 | -0.072 ± 0.178 | -0.118 ± 0.164 | -0.112 ± 0.171 | <0.001 | NS | NS |
| Visual-CinguloOpercular | 0.091 ± 0.104 | -0.000 ± 0.102 | -0.013 ± 0.138 | -0.024 ± 0.137 | <0.001 | NS | NS |
| Visual-Dorsal Attention | 0.014 ± 0.122 | -0.053 ± 0.115 | -0.087 ± 0.130 | -0.085 ± 0.134 | <0.001 | NS | NS |
| Visual-Ventral Attention | 0.087 ± 0.139 | 0.045 ± 0.107 | -0.020 ± 0.159 | 0.001 ± 0.138 | <0.001 | 0.024 | NS |
| Visual-Salience | 0.009 ± 0.183 | -0.085 ± 0.169 | -0.096 ± 0.203 | -0.084 ± 0.233 | <0.001 | NS | NS |
| FrontoParietal-CinguloOpercular | 0.018 ± 0.197 | -0.013 ± 0.179 | -0.084 ± 0.198 | -0.075 ± 0.216 | <0.001 | 0.046 | NS |
| FrontoParietal-Somatomotor | -0.064 ± 0.162 | -0.109 ± 0.155 | -0.161 ± 0.162 | -0.125 ± 0.161 | <0.001 | NS | <0.001* |
| FrontoParietal-Ventral Attention | 0.106 ± 0.206 | 0.105 ± 0.199 | 0.001 ± 0.209 | 0.024 ± 0.213 | <0.001 | 0.011* | NS |
| FrontoParietal-CinguloParietal | 0.097 ± 0.207 | 0.088 ± 0.210 | -0.018 ± 0.221 | 0.018 ± 0.199 | <0.001 | 0.027 | 0.014 |
| FrontoParietal-Auditory | 0.097 ± 0.207 | 0.088 ± 0.210 | -0.097 ± 0.180 | -0.085 ± 0.183 | <0.001 | NS | NS |
| CinguloOpercular-Salience | 0.236 ± 0.168 | 0.218 ± 0.196 | 0.113 ± 0.204 | 0.137 ± 0.201 | <0.001 | 0.007* | NS |
| CinguloOpercular-Dorsal Attention | 0.515 ± 0.231 | 0.460 ± 0.225 | 0.378 ± 0.247 | 0.401 ± 0.241 | <0.001 | NS | NS |
| Salience-Dorsal Attention | 0.183 ± 0.174 | 0.156 ± 0.209 | 0.073 ± 0.208 | 0.084 ± 0.190 | <0.001 | 0.015* | NS |
| Salience-Auditory | 0.104 ± 0.187 | 0.037 ± 0.195 | -0.016 ± 0.234 | -0.016 ± 0.229 | <0.001 | NS | NS |
| Salience-Somatomotor | -0.010 ± 0.182 | -0.024 ± 0.217 | -0.120 ± 0.216 | -0.102 ± 0.207 | <0.001 | 0.014* | NS |
| Ventral Attention-Auditory | 0.395 ± 0.258 | 0.365 ± 0.236 | 0.253 ± 0.259 | 0.272 ± 0.238 | <0.001 | 0.031 | NS |
| Visual-unspecified | 0.054 ± 0.097 | 0.008 ± 0.098 | -0.06 ± 0.106 | -0.044 ± 0.112 | <0.001 | 0.002* | NS |

**S5: Replication of main connectome analyses using the AAL parcellation scheme**

The specific choice of a parcellation scheme can impact the results of a network analysis ([8-10](#_ENREF_8)). To ensure that our findings are robust to the choice of a particular parcellation scheme, we also tested our findings using the AAL atlas which is an anatomical based parcellation and different from the 333-region parcellation which is defined based on areas that have functional coherent resting state functional connectivity patterns. As done for the main analysis, resting fMRI timeseries were extracted for each of the AAL regions and inter-regional correlations were performed to obtain a 90 x 90 inter-regional connectivity matrix for every individual.

We performed two analyses: 1) First, we compared the MDD-R and MDD-NR groups for the whole 90x90 functional connectivity matrices using NBS analysis. 2) Secondly, we mapped each of the AAL regions on to each of the 333 functional parcellation regions and identified all the links from the 333x333 connectome matrix which corresponded to those that were significant in the AAL based NBS analysis in 1. We then used a multivariate ANOVA analysis to test which of these links were significantly different between Remitters and Non-Remitters. This approach serves to directly test the replicability of results across parcellation schemes and also labels the specific sub-regions of the AAL regions into the intrinsic functional networks.

***Results:*** 1) For the first NBS analysis using the AAL parcellation, a connectomic signature comprising of 22 edges connecting 21 nodes was significantly different in pre-treatment resting connectivity between MDD-R and MDD-NR groups (as observed for the functional parcellation MDD-R>MDD-NR; p=0.038 corrected for multiple comparisons; Supplementary Figure S3 & Supplementary Table S3). Qualitatively, the connectomic signatures from both parcellation schemes appear very similar and as observed for the main analysis, intra-network and inter-network connections related to the DMN and FPN were significantly different between MDD-R and MDD-NR groups.

2) For the second analysis that mapped each of AAL regions on to the functional parcellation regions, a total 285 links were identified which corresponded to those in the identified AAL based network. Of these, 147 links were significant with MDD-R>MDD-NR with 20/22 edges from the AAL based connectome analysis found to replicate. These data suggest that our findings were robust to choice of parcellation scheme.

**Supplementary Figure S3:** **Pre-treatment functional connectivity networks differentiating MDD-R from MDD-NR groups using the AAL atlas.** Colors of the AAL nodes represent the intrinsic brain networks that they belong to from mapping onto the functional parcellation atlas.

Abbreviations: DMN, default mode network; FPN, fronto-parietal network; SM, somatomotor; VAN, ventral attention network; DAN, dorsal attention network; CON, cingulo-opercular network; L, left; R, right


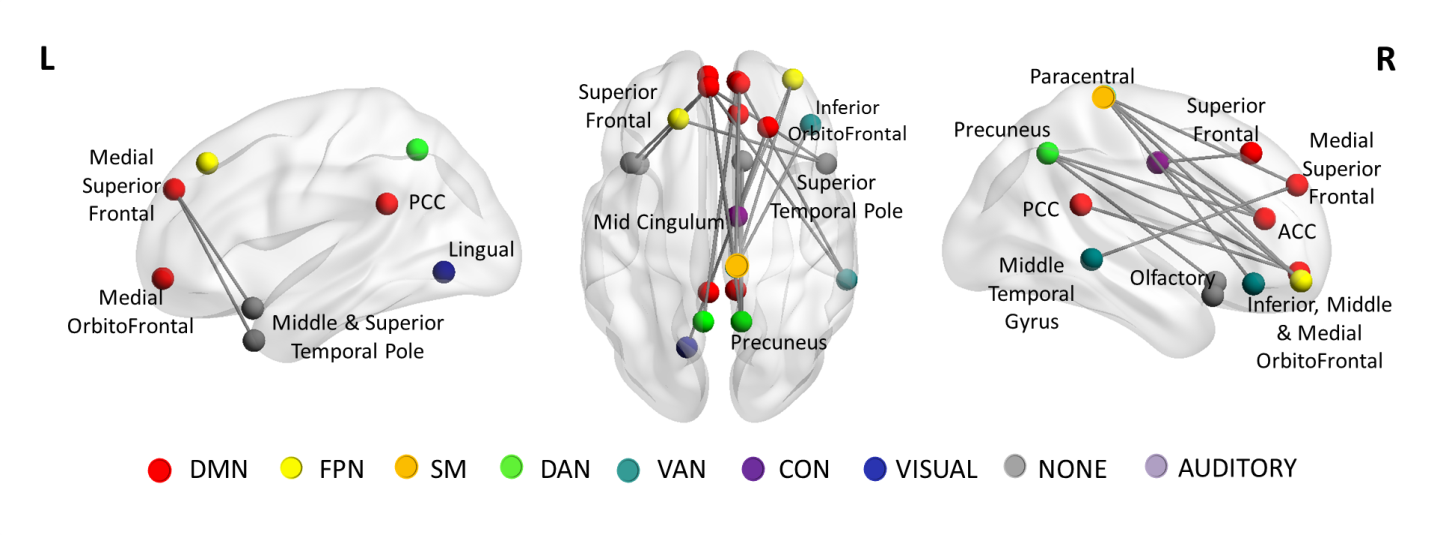


**Supplementary Table S3. Pretreatment network identified to be significantly different between MDD-R and MDD-NR groups using network-based statistical analysis of AAL atlas.** Mean and standard deviations for the resting functional connectivity estimates for the different connections are summarized. Intrinsic brain networks associated with each connection identified from the functional parcellation are also listed.

*Abbreviations:* L, left; MDD, major depressive disorder; R, right;

|  |  |  |  |  |
| --- | --- | --- | --- | --- |
| **Significant Network Connections (AAL Regions)** |  | **Resting fMRI connectivity** | | **p-value** |
|  | **Associated intrinsic brain networks.** | **MDD-R** | **MDD-NR** |  |
|  |  | **(N=58)** | **(N=105)** |  |
|  |  | **(Mean ± SD)** | **(Mean ± SD)** |  |
| Superior Frontal (R) to Mid Cingulum (R) | DMN (dmPFC) to DMN & CinguloOpercular | 0.39 ± 0.21 | 0.27 ± 0.21 | 0.001 |
|  | FrontoParietal (DLPFC) to DMN & CinguloOpercular |  |  |  |
| Middle Orbito-Frontal (R) to Mid Cingulum (R) | FrontoParietal to DMN | 0.08 ± 0.18 | -0.03 ± 0.23 | 0.002 |
|  | FrontoParietal to CinguloOpercular |  |  |  |
| ACC (R) to Mid Cingulum (R) | DMN to DMN & CinguloOpercular | 0.42 ± 0.21 | 0.30 ± 0.23 | 0.001 |
|  | Salience to DMN & CinguloOpercular |  |  |  |
| Medial Orbito-Frontal (R) to PCC (L) | DMN (vmPFC) to DMN | 0.51 ± 0.22 | 0.37 ± 0.28 | 0.001 |
| Medial Orbito-Frontal (R) to PCC (R) | DMN (vmPFC) to DMN | 0.44 ± 0.21 | 0.28 ± 0.28 | <0.001 |
| Superior Frontal (R) to Lingual gyrus (L) | DMN (dmPFC) & FrontoParietal (DLPFC) to Visual | -0.04 ± 0.19 | -0.17 ± 0.26 | 0.001 |
| Medial Orbito-Frontal (R) to Precuneus (L) | DMN (vmPFC) to DMN; CinguloParietal & Dorsal Attention | 0.30 ± 0.21 | 0.15 ± 0.33 | 0.001 |
| Olfactory (R) to Precuneus (R) | Subcallosal with DMN | 0.05 ± 0.17 | -0.08 ± 0.27 | 0.002 |
| Medial Orbito-Frontal (L) to Precuneus (R) | DMN (sgACC/vmPFC) to DMN & Dorsal Attention | 0.23 ± 0.20 | 0.09 ± 0.31 | 0.001 |
| Medial Orbito-Frontal (R) to Precuneus (R) | DMN (vmPFC) to DMN & Dorsal Attention | 0.24 ± 0.19 | 0.06 ± 0.34 | <0.001 |
| ACC (R) to Precuneus (R) | DMN to DMN; Dorsal Attention | 0.19 ± 0.22 | 0.04 ± 0.28 | 0.001 |
|  | Salience to DMN, Dorsal Attention |  |  |  |
| Middle Orbito-Frontal (R) to Paracentral Lobule (R) | FrontoParietal to Somatomotor | 0.11 ± 0.23 | -0.04 ± 0.29 | 0.001 |
| Inferior Orbito-Frontal (R) to Paracentral Lobule (R) | FrontoParietal & Ventral Attention to Somatomotor | 0.17 ± 0.19 | 0.03 ± 0.28 | 0.001 |
| Medial Superior Frontal (L) to Paracentral Lobule (R) | DMN & FrontoParietal (both dmPFC) to Somatomotor | 0.13 ± 0.24 | -0.01 ± 0.27 | 0.001 |
| Medial Superior Frontal (R) to Paracentral Lobule (R) | DMN & FrontoParietal (both dmPFC) to Somatomotor | 0.14 ± 0.23 | -0.03 ± 0.28 | <0.001 |
| ACC (R) to Paracentral Lobule (R) | DMN & Salience to Somatomotor | 0.02 ± 0.22 | -0.10 ± 0.24 | 0.001 |
| Medial Superior Frontal (L) to Superior Temporal Pole (L) | DMN (dmPFC) & FrontoParietal to Temporal | 0.35 ± 0.26 | 0.20 ± 0.27 | 0.001 |
| Superior Frontal (L) to Superior Temporal Pole (R) | *NA | 0.06 ± 0.24 | -0.08 ± 0.25 | 0.001 |
| Medial Superior Frontal (L) to Superior Temporal Pole (R) | *NA | 0.13 ± 0.23 | -0.01 ± 0.27 | 0.001 |
| Medial Superior Frontal (L) to Middle Temporal Gyrus (R) | DMN (dmPFC) to DMN & Ventral Attention | 0.38 ± 0.26 | 0.24 ± 0.28 | 0.002 |
| Medial Superior Frontal (R) to Middle Temporal Gyrus (R) | DMN (dmPFC) to DMN & Ventral Attention | 0.49 ± 0.24 | 0.33 ± 0.32 | 0.001 |
|  | FrontoParietal (dmPFC) to Ventral Attention |  |  |  |
| Medial Superior Frontal (L) to Middle Temporal Pole (L) | DMN (dmPFC) & FrontoParietal to Temporal | 0.33 ± 0.22 | 0.21 ± 0.24 | 0.001 |
|  |  |  |  |  |
| *Network links that did not replicate with the functional parcellation | |  |  |  |

**References:**

1. Korgaonkar MS, Grieve SM, Etkin A, Koslow SH, Williams LM. Using standardized fMRI protocols to identify patterns of prefrontal circuit dysregulation that are common and specific to cognitive and emotional tasks in major depressive disorder: first wave results from the iSPOT-D study. Neuropsychopharmacology : official publication of the American College of Neuropsychopharmacology. 2013;38(5):863-71.

2. Power JD, Barnes KA, Snyder AZ, Schlaggar BL, Petersen SE. Spurious but systematic correlations in functional connectivity MRI networks arise from subject motion. NeuroImage. 2012;59(3):2142-54.

3. Power JD, Mitra A, Laumann TO, Snyder AZ, Schlaggar BL, Petersen SE. Methods to detect, characterize, and remove motion artifact in resting state fMRI. NeuroImage. 2014;84:320-41.

4. Siegel JS, Power JD, Dubis JW, Vogel AC, Church JA, Schlaggar BL, et al. Statistical improvements in functional magnetic resonance imaging analyses produced by censoring high-motion data points. Human brain mapping. 2014;35(5):1981-96.

5. Andersson JL, Jenkinson M, Smith SM. Non-linear optimisation. FMRIB technical report tr07ja1.

6. Friston KJ, Williams S, Howard R, Frackowiak RS, Turner R. Movement-related effects in fMRI time-series. Magnetic resonance in medicine. 1996;35(3):346-55.

7. Korgaonkar MS, Ram K, Williams LM, Gatt JM, Grieve SM. Establishing the resting state default mode network derived from functional magnetic resonance imaging tasks as an endophenotype: A twins study. Human brain mapping. 2014;35(8):3893-902.

8. Fornito A, Zalesky A, Bullmore ET. Network scaling effects in graph analytic studies of human resting-state FMRI data. Frontiers in systems neuroscience. 2010;4:22.

9. Wang J, Wang L, Zang Y, Yang H, Tang H, Gong Q, et al. Parcellation-dependent small-world brain functional networks: a resting-state fMRI study. Human brain mapping. 2009;30(5):1511-23.

10. Zalesky A, Fornito A, Harding IH, Cocchi L, Yucel M, Pantelis C, et al. Whole-brain anatomical networks: does the choice of nodes matter? NeuroImage. 2010;50(3):970-83.
